# Gene therapy for the heart: encapsulated viruses to the rescue

**DOI:** 10.20517/evcna.2023.70

**Published:** 2024-02-26

**Authors:** Uma Maheswari Deshetty, Susmita Sil, Shilpa Buch

**Affiliations:** Department of Pharmacology and Experimental Neuroscience, University of Nebraska Medical Center, Omaha, NE 68198-5880, USA

**Keywords:** Extracellular vesicle, AAVs, genetic therapy, heart diseases

## Abstract

This commentary provides an in-depth analysis and perspective on the pioneering research article titled ‘Extracellular Vesicle-Encapsulated Adeno-Associated Viruses for Therapeutic Gene Delivery to the Heart’. The original study explores the innovative use of extracellular vesicle-encapsulated AAVs (EV-AAV-6 and -9) as a superior gene-delivery approach for cardiomyocytes (CMs), which not only provides increased AAV neutralizing antibody (NAb) resistance but also has implications for increased gene delivery efficacy to ischemic hearts. This study examined the efficacy of EVs isolated from the conditioned medium of AAV-6 and -9 producing HEK293T cells in combinatorial *in vitro* and in *vivo model* systems in comparison to free AAVs in the presence of the NAbs. This commentary highlights the key findings, discusses potential implications, limitations, and suggests future directions for research in this evolving field.

Heart disease is the leading cause of death in the United States. Cellular therapies have emerged as promising modalities for patients afflicted with heart disease^[1,2]^. Extracellular vesicles (EVs) - nano-sized (30-200 nm) cell membrane-derived spherical structures, released by various cell types, are known to shuttle bioactive cargoes to recipient cells. In recent times, EVs have been increasingly leveraged for therapeutic use in the treatment of several diseases^[3,4,5]^. Along these lines, an elegant study by Cambier *et al.* demonstrated the efficacy of cardiosphere-derived EVs carrying the Y-RNA (EV-YF1), in reducing the size of the myocardial infarct via modulation of interleukin (IL)-10 expression^[6]^. In addition, several other myocardial gene transfer strategies have also been developed utilizing the Adeno-Associated Vectors (AAVs), primarily owing to their attributes of non-pathogenicity and enhanced transgene expression^[7-13]^. A key limitation in the success of AAV strategies, however, has been the presence in the host of the AAV neutralizing antibodies (NAbs), which negates the efficacy of AAV gene therapy^[14-17]^. To circumvent this roadblock, recent years have seen the use of EV-entrapped AAVs as a promising gene therapy approach for counteracting the effects of AAV NAbs. While such approaches have been developed, one caveat with these has been the presence of free AAVs, which are also secreted along with the EV-AAVs from the conditioned media of the AAV-producing cells. The presence of these free AAVs, in turn, results in decreased EV-AAV dosing, lowering their NAb resistance while also inducing deleterious side effects of free AAVs. There is thus an urgent need to utilize highly purified and characterized EV-AAVs devoid of free AAVs for gene therapy approaches.

The study by Li *et al.* makes a pivotal contribution to the field of gene therapy by demonstrating the use of purified EV-AAVs as potential cardiac gene therapeutic carriers for enhancing long-term gene delivery to the heart. This study provides the functional benefits and mechanisms underlying the EV-AAV approach. Herein, the authors optimized the EV-AAV purification strategy, and characterized in-depth the EV-AAVs with validation in five different *in vitro* and *in vivo* model systems, establishing cardiotropism and higher uptake and therapeutic efficacy of this vector. Specifically, the authors tested the efficacy of delivering SERCA2A as a therapeutic target via EV-AAVs (AAV-6 and AAV-9) both in the human left ventricular CMs as well as induced human pluripotent stem cell (hiPSC)-derived CMs. This was also validated *in vivo* in murine hearts, wherein the therapeutic potential of EV-AAV-SERCA2A was compared with the AAV-SERCA2A delivery in a preimmunized mouse model of myocardial infarction. This study effectively demonstrated that EV-AAV-SERCA2A not only effectively transduced the therapeutic gene into the CMs, despite the presence of NAbs, but that such an approach was also effective in improving the cardiac function, and remodeling in mice with myocardial infarct. The present study emphasizes the potential clinical translation of such an approach for AAV NAb^+^ patients with heart failure, even in treated patients. This sets the stage for future refinement and development of therapeutic, adjunctive therapies for heart failure patients with AA NAbs^[18]^. This study also delineated the uptake of EV-AAVs into the acidic subcellular compartments such as late endosomes and lysosomes, which, in turn, could release the AAVs from EV-AAVs, and thus facilitate nuclear entry and gene expression.

While the authors have also looked at the non-cardiomyocyte cells, one of the limitations of this study is the possible off-target effects of such an approach. Despite the advantages of AAV/EV-AAV vectors in localized intramyocardial delivery, it is not fraught without off-target delivery to organs such as the liver. The use of engineered AAVs88 containing EV-AAVs, which possess enhanced cardiac or muscle delivery potency, could thus prove valuable as an effective approach for translational studies. Although the authors demonstrated that EV-AAVs expressed increased numbers of transgenes in CMs compared to non-cardiomyocytes (NMs), it would have behooved the authors to provide some discussion on the possible off-target effects of such an approach, specifically for clinical translational ramifications. Additionally, as rightly pointed out by the authors, since AAVR could likely be a common receptor for both AAVs and EV-AAVs, it would be necessary for EV-AAVs to undergo further purification to remove traces of AAV contaminants prior to moving this approach into preclinical and/or clinical applications. It would have been important for the authors to have also included the rationale for the choice of AAV-6 over AAV-9 in this study^[18]^. Furthermore, there is no mention of how the EVs from HEK cells can target CMs efficiently. One speculates if there is a specificity of the HEK EVs for CMs or that just a small proportion of the injected EVs actually reach the heart. It is essential to understand what proportion of EVs reach the heart. The authors’ thoughts on the biodistribution of these EVs, implications for targeted therapy, and the next steps critical for moving the product into the clinical area would benefit the readers.

Future studies should be aimed at examining the efficacy of various routes of delivery of these therapeutic EVs. Understanding the half-life of these therapeutic EVs and the stability of the gene expression in an organism would be the next step before embarking on human clinical trials. Combining the delivery of several other target genes would also be the next step as an effective delivery approach.

Overall, the research article marks a significant advancement in the field of gene therapy by introducing the integration of two powerful vector systems - EV & AAVs and holds promise for addressing longstanding challenges associated with traditional AAV delivery methods. Although, current research suggests that small EVs secreted from the AAV-producing HEK293T cells can effectively deliver intact AAVs in several organ systems such as the nervous system, inner ear, retina, and liver in mice^[19-23]^, the production of pure EV-AAVs devoid of free AAVs is the need of the hour to minimize NAb effect. Additionally, while the previous studies targeted the non-cardiac tissues (retina and brain)^[21-25]^, the use of CM-targeting strategy via EV-AAVs with improved delivery of AAVs in the cardiac muscle is an important outcome in this article, leading a step forward towards clinical translatio^[26,27]^. As summarized in Figure 1, EV-AAVs were identified as potent delivery vectors for targeted cardiac gene therapy by using five *in vitro* and *in vivo* methods. In conclusion, authors reported enriched isolation, and characterization of purified EV-AAVs with their validation as an effective therapeutic modality for improvement of myocardial infarction outcome with minimal Nab effects. Future studies aimed at testing the efficacy of this approach in humans by including other gene therapy targets will be of value. Overall, this is an innovative approach that has the potential to revolutionize gene delivery strategies and unlock new horizons for the treatment of various pathologies.

**Figure 1:**
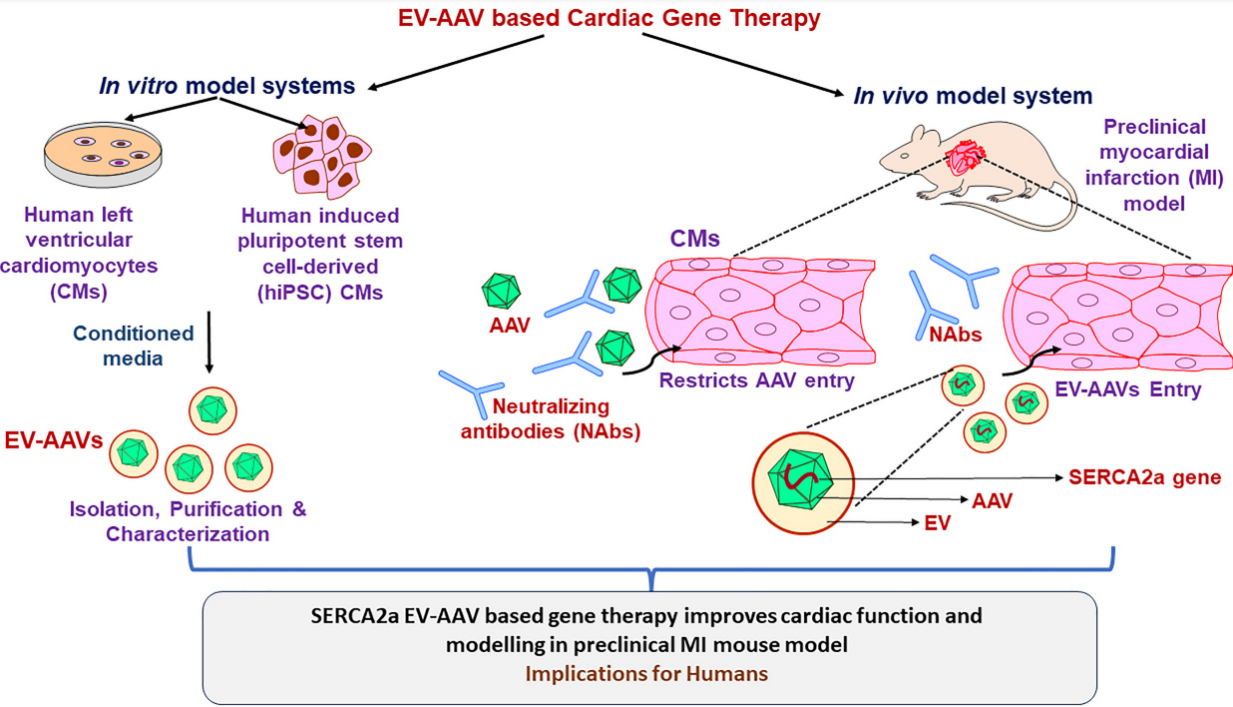
: Schematic for EV-AAV-based cardiac gene therapy using *in vitro* and *in vivo* models.

## DECLARATIONS

### Copyright

© The Author(s) 2024.
